# Effects of intravenous furosemide plus small-volume hypertonic saline solutions on inflammatory, remodelling markers and epigenetics signatures of patients with congestive acute decompensated heart failure (ADHF)

**DOI:** 10.18632/aging.206364

**Published:** 2026-03-26

**Authors:** Mario Daidone, Alessandra Casuccio, John Sebastian Soldano, Valerio Vassallo, Gaetano Pacinella, Maria Grazia Puleo, Roberta Oliveri, Giuseppe Clemente, Daniela Colomba, Giuseppe Miceli, Vittoriano Della Corte, Rosaria Pecoraro, Tiziana Di Chiara, Domenico Di Raimondo, Carlo Domenico Maida, Sergio Ferrantelli, Antonino Tuttolomondo

**Affiliations:** 1 Internal Medicine and Stroke Care Ward, University Hospital, Policlinico, Paolo Giaccone, 90127 Palermo, Italy; 2 Department of Health Promotion, Mother and Child Care, Internal Medicine and Medical Specialties, University of Palermo, 90127 Palermo, Italy; 3 Molecular and Clinical Medicine PhD Program, University of Palermo, 90127 Palermo, Italy

**Keywords:** heart failure, acute decompensated heart failure, furosemide, hypertonic saline solution

## Abstract

Aims: In a randomised controlled trial (RCT), we compared the effects of treatment with furosemide + small volumes of hypertonic saline solution (HSS) with those of furosemide alone in patients with decompensated heart failure (HF), and their effects on inflammatory and remodelling markers and epigenetic signatures.

Methods: All consecutive patients with acute decompensated heart failure (ADHF) due to heart failure with reduced ejection fraction (HFrEF) were enrolled. Patients were randomly assigned to treatment with i.v. furosemide plus HSS or i.v. furosemide alone. Patients were evaluated at T0 (admission), T1 (after treatment), and T2 (after a saline bolus) to determine serum concentrations of NT-proBNP, hsTnT, s-ST2, galectin-3, IL-6, and CRP and to evaluate some selected miRNA concentrations.

Results: We enrolled 200 subjects, 107 randomized to the furosemide plus HSS, and 93 to furosemide alone. At T1, patients treated with high-dose furosemide + HSS had higher absolute delta values of IL-6, hsTnT, NT-proBNP and galectin-3. Patients treated with i.v. furosemide + HSS showed significantly lower increases in the serum concentrations of IL-6, hsTNT, sST2, galectin-3 and NT-proBNP after saline load. We observed a decrease in miR181b expression in subjects treated with i.v. furosemide plus HSS in comparison to patients treated with i.v. furosemide alone and a more significant reduction of miRNA181b expression in subjects treated with furosemide plus HSS.

Conclusions: Our findings revealed that in subjects with ADHF, treatment with i.v. furosemide plus HSS significantly decreased the serum levels of IL-6, sST2, hsTnT, galectin-3, and NT-proBNP and modulated some miRNA expression.

## INTRODUCTION

Heart failure (HF) is one of the principal causes of mortality in industrialized countries, particularly in ageing populations. Advances in coronary revascularization techniques have contributed to a decline in deaths from acute myocardial infarction; however, they have also led to a progressive rise in the prevalence of post-ischemic HF. In contrast to the substantial progress in revascularization strategies, genuinely innovative clinical approaches aimed at targeting the wound-healing phase of myocardial injury and favorably modulating cardiac remodeling remain relatively limited [1, 2].

HF is a complex multisystem syndrome characterized by impaired cardiac performance. A reduction in cardiac output, largely due to left ventricular dysfunction, triggers compensatory activation of neurohormonal pathways. This, in turn, stimulates the renin–angiotensin–aldosterone system, resulting in increased levels of renin, angiotensin II and aldosterone, which promote sodium and water retention, vasoconstriction and heightened sympathetic drive. Chronic exposure to this neurohormonal activation induces ventricular dilatation, structural myocardial remodeling and fibrosis, thereby further compromising cardiac output [3, 4].

The HF syndrome encompasses both microstructural and macrostructural alterations, each accompanied by activation of inflammatory and neurohormonal cascades that release a wide array of biomolecules in an attempt to compensate for the failing heart. As a consequence, a “storm” of cytokines and regulatory mediators is generated. The sheer number and dysregulation of these molecules make it challenging to identify specific biomarkers that are closely linked to the clinical setting of acute decompensated heart failure (ADHF).

These dysregulated molecules may serve in clinical and scientific practice as potential remodeling markers such as suppression of tumorigenicity 2 (ST2), Galectin-3mm, troponin-T, or potential cardiac stretching markers such as NT-proBNP, or inflammatory markers such as some proinflammatory cytokines. Furthermore, the epigenetic alterations such as the microRNAs expression changes occurring in the clinical context of the heart failure syndrome may be used as epigenetic signatures of heart failing.

In response to mechanical strain, cardiomyocytes synthesize suppression of tumorigenicity 2 (ST2), which is present in two isoforms: a transmembrane/cellular form (ST2L) and a soluble circulating form (sST2) [5]. ST2 functions as the receptor for interleukin-33 (IL-33), a cytokine released following cellular injury and predominantly expressed by endothelial and epithelial cells [6]. Elevated levels of sST2 act as a decoy receptor, sequestering IL-33 and thereby limiting its interaction with ST2L; this mechanism attenuates the cardioprotective effects of IL-33 observed in experimental models, including the reduction of myocardial fibrosis, cardiomyocyte hypertrophy and apoptosis [7].

Galectin-3, predominantly produced by macrophages but also expressed by other cell types, promotes fibroblast proliferation and stimulates collagen deposition within the myocardium [8, 9]. Several studies have demonstrated that increased circulating Galectin-3 levels are associated with newly diagnosed HF and that this biomarker provides additional value as an indicator of prognosis and disease severity in patients with both HFrEF and HFpEF [10, 11].

Troponin is a regulatory protein complex of the sarcomere, composed of three distinct subunits (I, C, and T). Elevated serum troponin concentrations correlate with greater disease severity and higher mortality risk, whereas decreasing levels are associated with a more favorable prognosis [12].

Natriuretic peptides (NPs) are synthesized in the heart in response to atrial and ventricular wall stretch and neurohormonal activation [13]. Both BNP (B-type natriuretic peptide) and NT-proBNP (N-terminal pro-B-type natriuretic peptide) are routinely employed to support diagnosis, monitor therapeutic efficacy, and estimate prognosis in patients with HFrEF [14].

Proinflammatory cytokines, including interleukin-6 (IL-6), interleukin-1 (IL-1) and tumor necrosis factor-alpha (TNF-α), are upregulated in individuals with HF, and their circulating levels show a direct relationship with both New York Heart Association (NYHA) functional class and left ventricular ejection fraction (EF) [15–17].

Circulating microRNA (miRNA) levels change dynamically in the setting of multiple acute and chronic conditions, and their remarkable stability in stored biological samples supports their potential as biomarkers in HF [18]. However, a clear agreement is still lacking regarding which specific serum miRNAs might represent the optimal markers for HF. Emerging data indicate that distinct miRNAs are differentially regulated in the failing myocardium, influencing several dimensions of the HF phenotype [19, 20, 21]. MiRNAs have been implicated in HF pathogenesis by negatively modulating the expression of genes that orchestrate adaptive and maladaptive cardiac remodeling. Prior investigations have documented profound changes in myocardial miRNA expression profiles in HF, while other studies have demonstrated their involvement in key signaling pathways implicated in disease progression [19, 22].

In a notable study, investigators explored whether left ventricular reverse remodeling after cardiac resynchronization therapy (CRT) in patients with HF and ventricular dyssynchrony was linked to modifications in circulating miRNAs [23]. They observed that responders exhibited higher expression of five miRNAs implicated in cardioprotection against adverse remodeling. The authors concluded that a favorable therapeutic response in terms of reverse remodeling is accompanied by beneficial shifts in miRNAs that regulate cardiac fibrosis, apoptosis and hypertrophy.

In both experimental models and human myocardium, it is generally accepted that alterations in cardiomyocyte biology represent the principal trigger for cardiac remodelling, although some experimental settings demonstrate that remodelling can develop even in the absence of overt myocyte dysfunction. Reverse remodelling, or cardiac de-remodelling, denotes the partial or complete reversal of disease- or injury-induced structural and functional changes, particularly in the context of HF. It is characterized by a reduction in chamber dimensions, especially of the left ventricle, together with improved contractile performance. This phenomenon is usually interpreted as a marker of myocardial recovery and is often a key therapeutic objective in HF management [24]. On the basis of current evidence, several microRNAs have been shown to regulate and/or modulate critical elements of the hypertrophic programme in cardiac myocytes, including reactivation of the so-called fetal gene pattern, and may also participate in processes of reverse remodelling [22, 23].

A number of miRNAs have been linked to pathological pathways involved in HF development and progression [21–23, 25]. Among these, miRNA-365 (miR-365) has emerged as an important member of the miRNA family, with several studies indicating a pivotal role in the evolution of pathological ventricular dysfunction. In an expression-profiling study, Jentzsch et al. [9] observed that miR-365 was markedly upregulated and positively correlated with the degree of myocardial hypertrophy, suggesting a potential pro-hypertrophic action of this miRNA [26].

The miR-125 family, and particularly miR-125a-5p, has also been implicated in HF, especially in the setting of myocardial infarction (MI). Experimental data indicate that miR-125a-5p exerts anti-apoptotic effects in cardiomyocytes, thereby conferring protection against ischemia-induced cell death. Furthermore, one study reported that miR-125a-5p expression patterns could discriminate HFREF from HFPEF [27].

More recently, miR-150-5p has been proposed as a novel circulating biomarker for AHF. Its association with maladaptive remodelling, disease severity and adverse outcomes supports the pathophysiological significance of reduced miR-150-5p expression in AHF [28].

MicroRNA-181b (miR-181b) has likewise been identified as both a potential biomarker and a putative therapeutic target in HF. Altered miR-181b levels have been reported in patients with HF, and accumulating data suggests that it contributes to the regulation of inflammatory responses and cardiac remodelling. A recent study further indicated that miR-181b may serve as a promising circulating biomarker for HF [29].

The circulating expression profile of miR-214 and its contribution to HF pathogenesis has been comparatively less explored. However, a recent investigation clarified the biological and clinical relevance of miR-214 dysregulation in HF. Mechanistically, miR-214 overexpression was shown to impair angiogenesis in HUVECs by targeting XBP1, a key transcription factor in the unfolded protein response; silencing XBP1 reproduced the reductions in HUVEC proliferation and angiogenic capacity observed with miR-214 overexpression. Collectively, these data indicate that miR-214 is a critical modulator of cardiac angiogenesis *in vitro* and *in vivo*, most likely through regulation of XBP1 expression, and support a central role for miR-214 in the control/inhibition of angiogenic processes in the failing heart [30].

Preliminary investigations [21, 25] have demonstrated that the use of an intravenous combination of hypertonic saline solution (HSS) and high-dose furosemide is both safe and well tolerated in patients with congestive HF. These studies were based on the hypothesis that preserving adequate intravascular volume and renal perfusion during high-dose loop diuretic therapy may potentiate its diuretic efficacy. Both objectives can be pursued by coupling high-dose furosemide with HSS administration.

In this context, our group previously showed that, in patients with ADHF, treatment with furosemide plus HSS, as compared with furosemide alone, led to a significant reduction in circulating ANP, BNP, TNF-α, IL-1β and IL-6 levels. We further reported that, following an 8-day course of the furosemide + HSS regimen, an acute saline challenge (15 mL/kg of 0.9% NaCl) induced a smaller relative increase in serum ANP, BNP, tumor necrosis factor and IL-1β in these patients compared with control groups [31, 32].

Further studies, more recently, showed the superiority of combination HSS plus furosemide therapy over furosemide alone in terms of kidney function preservation, improved diuresis and natriuresis, weight loss, duration of hospital stay and mortality confirming that in both hospitalized and ambulatory patients with heart failure syndrome, adding HSS to furosemide may improve short-term natriuretic response and outcomes [33–37].

Nevertheless, the possible effects of the i.v. furosemide + HSS treatment on natriuretic and inflammatory markers of heart failure deserve further confirmation, whereas the effects of this type of treatment on epigenetic signatures of pathologic mechanisms involved in the left ventricular dysfunction involved in AHF pathogenesis seem to be still not studied.

### Study hypothesis

In this study, we aimed to assess the impact of moderate-to-high doses of intravenous furosemide combined with small volumes of HSS on circulating HF-related biomarkers— NT-proBNP, hsTnT, galectin-3, IL-6, ST2 and CRP—in patients with ADHF secondary to HFrEF.

We hypothesized that, compared with i.v. furosemide alone, the furosemide + HSS regimen would induce a greater reduction in serum concentrations of these HF biomarkers after decongestive treatment and lead to a smaller rise in their levels following an acute saline challenge performed in a compensated phase, reflecting partial or complete resolution of congestion. This effect was presumed to result from a more favorable modulation of myocardial stretch and inflammatory burden afforded by the addition of HSS to the standard i.v. furosemide protocol.

We also aimed to address further the yet actual knowledge gap concerning the role of miRNA not only as a pathogenetic factor but also in the context of a hypothesis that epigenetic signatures such as microRNA change fold may serve as possible therapeutic efficacy markers. Thus, we investigated the effects of treatment with high dose furosemide + HSS on fold changes of circulating miRNAs potentially involved in structural alterations of the failing heart.

We have chosen some miRNA as indicated above in the introduction section for their reported pathogenetic or protective role in heart failure.

### Aims of the study

We sought to evaluate in a randomized clinical trial, the efficacy (primary outcome) of treatment with i.v. furosemide + HSS by comparing the reduction degree of serum levels of some chosen markers of HF and the modulation response degree of chosen miRNAs, as secondary outcome, after treatment with i.v. furosemide plus HSS and in a compensated state after an acute saline load compared to treatment with i.v. furosemide alone.

## MATERIALS AND METHODS

We prospectively enrolled all consecutive patients older than 18 years admitted to the Internal Medicine Unit of Policlinico “P. Giaccone” in Palermo with a diagnosis of ADHF due to HFrEF between March 2019 and December 2021. Eligible patients were randomly allocated, in a 1:1 ratio, to receive either moderate/high doses of i.v. furosemide in combination with HSS or i.v. furosemide alone, according to a computer-generated randomization list.

### Study protocol

Patients were assigned to the i.v. furosemide + HSS arm or to the i.v. furosemide-only arm using a computer-generated sequence (1:1). Each participant underwent three study assessments: at T0 (on admission, before initiation of moderate/high-dose furosemide + HSS or furosemide alone), at T1 (after six days of the assigned i.v. therapy), and at T2 (after the saline load). At each time point, venous blood samples were obtained for measurement of NT-proBNP, hsTnT, sST2, galectin-3, IL-6 and CRP.

### Inclusion criteria

All consecutive patients admitted to our Internal Medicine ward between March 2019 and December 2021 with a diagnosis of ADHF and HFrEF were considered for enrolment.

### Exclusion criteria

Patients were excluded if they had acute myocarditis, active pulmonary or liver disease, autoimmune disorders, ongoing infection, malignant disease, muscle disease, renal insufficiency (serum creatinine ≥2.5 mg/dL), chronic inflammatory or rheumatologic conditions, haematological disorders, or if they were receiving regular anti-inflammatory therapy.

### Disease definitions

HF was defined according to European Society of Cardiology criteria, i.e., the presence of typical symptoms and/or signs of congestive HF (New York Heart Association functional class II or higher) together with objective evidence of cardiac dysfunction [28]. ADHF was defined as an acute clinical presentation characterized by signs and symptoms of congestion and fluid retention (weight gain, exertional dyspnoea, orthopnoea, dependent oedema) in the setting of established congestive HF [28]. HFrEF was defined, in line with ESC guidelines, as HF with a left ventricular ejection fraction <40% [28].

The aetiology of HF was determined from clinical history and medical records, including prior diagnoses of hypertension, myocardial infarction, angina, valvular heart disease, diabetes or non-ischaemic dilated cardiomyopathy.

The study protocol was approved by the Ethics Committee of Policlinico “P. Giaccone” of Palermo, Italy, and written informed consent was obtained from all participants. The trial was registered at ClinicalTrials.gov (Identifier: NCT04628325).

### Clinical and laboratory evaluation

In both the “furosemide/HSS” and “furosemide alone” groups, body weight and 24-hour urine volume were recorded daily. Fasting blood samples were obtained each day during hospitalization to determine serum Na, K, Cl, albumin, uric acid, creatinine, urea and glucose, and monitoring continued until a clinically compensated state was achieved at the end of the treatment period. Daily urine collections were used to quantify diuresis and to measure urinary Na, K and Cl excretion.

### Randomization

Following a 2-week placebo screening and run-in phase, patients were randomized in a 1:1 fashion to Group A (furosemide/HSS) or Group B (furosemide alone) using a computer-generated random sequence (IBM SPSS Software, version 24). Allocation codes were placed in sequentially numbered, opaque, sealed envelopes, each containing the treatment assignment for a single patient. The randomization list was prepared by a biostatistician, while investigators were responsible for patient enrolment. The randomization code was held centrally and was not disclosed until completion of data analysis. Investigators involved in clinical data collection and outcome measurements were not directly involved in the administration of the study treatments and were masked to the randomization process.

### Treatment protocol

Patients assigned to the furosemide + HSS group received a 30-minute intravenous infusion twice daily for 6 days from admission, consisting of furosemide (120–250 mg) combined with HSS (150 mL of 1.4–4.6% NaCl). The daily furosemide dose was titrated according to urine output, blood pressure, and the severity of congestive signs and symptoms. The HSS concentration was individualized according to the following scheme:

- in patients with serum sodium <135 mEq/L, a 3.5% HSS solution was administered;
- in those with serum sodium >135 mEq/L, HSS concentration ranged empirically between 1.4% and 2.4% according to serum Na levels;
- KCl (20–40 mEq i.v.) was co-administered to prevent hypokalaemia.

Patients in the furosemide-only group received a 30-minute i.v. infusion of furosemide (120–250 mg) twice daily for 6 days from admission, without HSS. As in the combined-treatment group, the daily furosemide dosage was adjusted on the basis of urinary volume, blood pressure values, and the clinical burden of congestion.

At the end of the 6-day treatment phase, intravenous furosemide was discontinued and replaced with oral furosemide (25–250 mg/day). The maintenance dose was established according to the degree of left ventricular dysfunction, NYHA class, presence of congestive symptoms and signs, and blood pressure.

Throughout the study period, patients in both the “furosemide/HSS” and “furosemide alone” arms received background therapy with ACE inhibitors, digitalis, and nitrates, as appropriate. Physical activity was kept moderate in patients with mild-to-moderate symptoms (NYHA class II), whereas bed rest was recommended in the presence of more severe symptoms (NYHA class III–IV).

All participants were instructed to follow a low-sodium diet for 10 days, with a daily intake of 1.61 g of Na (70 mmol/day), corresponding to approximately 4.0 g of NaCl/day.

### Acute saline load

On the day after completion of the 6-day treatment phase, all participants in both the furosemide + HSS arm and the furosemide-only arm underwent an acute volume challenge consisting of an infusion of 15 mL/kg of 0.9% NaCl over 60 minutes, according to a protocol previously described by our group (25).

### Blood sample collection

Venous blood was drawn after at least 30 minutes of rest in the supine position. Sampling was performed within the first 24 hours after admission (T0), at the end of the 6-day treatment period (T1), and 24 hours after completion of the acute saline load given at the end of therapy with i.v. furosemide + HSS or i.v. furosemide alone (T2). At each time point (T0, T1 and T2), blood was collected to measure serum ST2, NT-proBNP, hsTnT, galectin-3, IL-6 and CRP.

### Laboratory procedures

Non-fasting venous blood samples were obtained by standard venipuncture. After centrifugation at 1700 g (relative centrifugal force), citrate, EDTA, heparin and Trasylol plasma fractions, as well as serum, were separated. Buffy coats from EDTA tubes were stored for subsequent genetic analyses. Dimethylsulfoxide was added to an additional EDTA tube to enable cryopreservation of blood cells. All aliquots were frozen at −80° C within 2 hours of collection.

### Galectin-3 assay

Serum galectin-3 concentrations were determined using the BGM Galectin-3 Test in accordance with the manufacturer’s instructions (BG Medicine, Inc., Waltham, MA, USA).

### Measurement of NT-proBNP

NT-proBNP levels were quantified in heparinized plasma with the Elecsys NT-proBNP assay on a Cobas 8000 platform (Roche Diagnostics Limited, Rotkreuz, Switzerland).

### Measurement of IL-6

Serum IL-6 was assessed by enzyme-linked immunosorbent assay (Intertest 6; Genzyme, Boston, MA) following the manufacturer’s protocol. The assay’s lower limit of detection was 76 pg/mL; values below this threshold were classified as undetectable.

### Measurement of CRP

CRP concentrations were measured using a fluorescence polarization immunoenzymatic method (Abbott Laboratories, Chicago, IL, USA), with a detection limit of 5 mg/L.

### Measurement of soluble ST2

Soluble ST2 was quantified using a sandwich double–monoclonal antibody ELISA (Medical and Biological Laboratories).

### RNA isolation

Total RNA was isolated from 100 μL of serum with the miRNeasy Serum/Plasma Advanced Kit (Qiagen), following the manufacturer’s protocol. Synthetic cel-miR-39-3p (1.6×10^8^ copies/μL; Qiagen) was added to each sample before extraction to allow normalization in downstream quantitative real-time PCR (qRT-PCR). RNA was eluted from the spin columns in 30 μL of water and subsequently treated with heparinase to remove residual heparin, which can interfere with PCR.

### qRT-PCR analysis of microRNAs

The extracted RNA was reverse transcribed to complementary DNA (cDNA) using the TaqMan MicroRNA Reverse Transcription Kit (Applied Biosystems, Carlsbad, CA, USA) according to the manufacturer’s instructions. qRT-PCR was performed on a Viia7 system (Thermo Fisher Scientific, Waltham, MA, USA) with specific TaqMan assays (Applied Biosystems) for miR-214, mir-365, mir-181b, miR-150-5p, miR-125a-5p. MiRNAs with Cq ≥36 were considered not detectable and were assigned a Cq value of 36. Relative miRNA abundance was calculated using the 2−^d^Cq approach (dCq = Cq[miR] − Cq[Cel-miR-]) and then transformed on a log_2_ scale.

- The primers used for miR-214 were: Forward primer: 5’-AGCATAATACAGCAGGCACAGAC-3’; Reverse primer: 5’-AAAGGTTGTTCTCCACTCTCTCAC-3’. The expression of PTEN mRNA was detected by quantitative PCR using paired primers. β-actin gene was used as control. The primers for PTEN mRNA were: Forward primer: 5’-ACCAGTGGCACTGTT GTTTCAC-3’; Reverse primer: 5’-TTCCTCTGGTCCTGGTATGAAG-3’.
- The primer sequences used for mir-365 were: miR-365 forward, 5′-GCGTAATGCCCCTAAAAATCC-3′, the reverse primer was universal and provided by the kit. The forward and reverse PCR primer sequences used for U6 were 5′-AACGCTTCACGAATTTGCGT-3′ and 5′-CTCGCTTCGGCAGCACA-3′, respectively.
- The primers used for mir-181b were: -5p: forward primer 5’-CCAGCTGGGCTCACTGAACAATGA-3’, reverse primer 5’-CAACTGGTGTCGTGGAGTCGGC-3’; *U6*: forward primer 5’-CTCGCTTCGGCAGCACA-3’, reverse primer 5’-AACGCTTCACGAATTTGCGT-3’; *SSX2IP*: forward primer 5’ CCGGGGAACTAAGCAGAGAGA-3’, reverse primer 5’-GTTCATGGTCTTGTCGTGAGAT-3’; *GAPDH*: forward primer 5’-GCACCGTCAAGCTGAGAAC-3’, reverse primer 5’-TGGTGAAGACGCCAGTGGA-3’.
- The primers used for miR-150-5p were: Forward: 5′-TCTCCCAACCCTTGTACCAGTG-3′, Reverse: 5′-GTGCGTGTCGTGGAGTC-3′
- The primers used for miR-125a-5p were: miR-125a-5p, forward 5′-TGAGACCCTTTAACCTGTGA-3′ and reverse 5′-GCGAGCACAGAATTAATACGAC-3′; U6, forward 5′-CTCGCTTCGGCAGCACA-3′and reverse 5′-AACGCTTCACGAATTTGCGT-3.

### Statistical analysis

The sample size was estimated to detect a 30% difference in mean serum concentrations of NT-proBNP, hsTnT, sST2, galectin-3, IL-6 and CRP between the two independent treatment groups, with 95% statistical power (β = 0.05), a 5% dropout rate and a two-sided α level of 0.05. This calculation indicated that at least 80 patients per group (160 subjects in total) were required.

Continuous variables are presented as mean ± standard deviation, unless otherwise specified. Baseline differences between the two groups (i.v. furosemide + HSS vs i.v. furosemide alone) were evaluated using Pearson’s chi-square test for categorical variables. When the assumptions for this test were not met (i.e., expected cell counts <5 in more than 20% of cells), Fisher’s exact test was applied. For continuous variables, distributional assumptions were checked with the Shapiro–Wilk test. Normally distributed variables were compared using one-way ANOVA, and, in the presence of statistically significant results, post hoc analyses were performed with Bonferroni correction for multiple testing. Variables that differed significantly between groups were further analyzed using analysis of covariance (ANCOVA), adjusting for hypertension, BMI, cholesterol and triglycerides. For each outcome, the effect of treatment group (1 vs 0) was estimated and reported as a point estimate (β for continuous variables and odds ratio for binary variables) with corresponding 95% confidence intervals and p-values.

To assess changes in continuous variables measured at multiple time points (e.g., microRNA expression at T0, T1 and T2), a repeated-measures ANOVA was conducted. When the assumption of sphericity was violated, as assessed by Mauchly’s test, the Greenhouse–Geisser correction was applied. Post hoc analyses for within-subject factors were carried out using pairwise comparisons with Bonferroni adjustment. All statistical analyses were performed using IBM SPSS Statistics, version 24 (IBM Corp., Armonk, NY, USA). All p-values were two-sided, and p ≤ 0.05 was considered statistically significant.

## RESULTS

We enrolled 216 patients with ADHF and HFrEF admitted to our Internal Medicine ward from March 2019 to December 2021.

Thirteen patients were excluded based on the presence of exclusion criteria. Three patients died during the study period. Thus, 107 patients were randomized to treatment with i.v. high-dose furosemide plus HSS, whereas 93 patients were randomized to i.v. high-dose furosemide alone.

General, demographic and laboratory variables in subjects treated with furosemide plus HSS versus furosemide alone group are listed in Table 1.

**Table 1:** General, demographic and laboratory variables in subjects treated with i.v. furosemide plus HSS vs subject group treated with i.v. furosemide alone.

	**Pts treated with i.v. furosemide plus HSS (n=107)**	**Pts treated with i.v. furosemide alone (n=93)**	**P**
**Age *(years) (mean±ds)***	75.4 ± 9.8	7 4.2 ± 6.3	0.332
**Sex *(M/F) (n/%)***	43/64	55/38	**0.011**
**SBP (mmHg) *(mean±ds)***	135.0 ± 22.04	120.5 ± 13.8	**<0.0005**
**DBP (mmHg) *(mean±ds)***	70.9 ± 9.4	71.6±12.06	0.668
**Weight (Kg) *(mean±ds)***	82.1± 14.2	85.4 ± 36.2	0.388
**BMI (Kg/m^2^)*(mean±ds)***	28.5 ± 5.6	25.7 ± 4.5	**<0.0005**
**WBC *(per microliter) (mean±ds)***	9485±3456	7573±2304	**<0.0005**
**Platelets** (**per microliter**) *(mean±ds)*	207394±146176	191043±44142	0.300
**RBC (per microliter) *(mean±ds)***	4165140±678615	4203064±662648	0.691
**Haemoglobin (g/*dl) (mean±ds)***	11.5±1.7	11.2±1.3	0.148
**Total cholesterol** (**mg/dl) *(mean±ds)***	134.2±35.9	106.1 ±74.2	**0.001**
**Triglycerides (*mg/dl) (mean±ds)***	93.3±35.3	64.9±26.4	**<0.0005**
**HDL cholesterol (mg/dl) *(mean±ds)***	41.7±12.4	44.6±17.7	0.177
**FPG (mg/dl) *(mean±ds)***	131 ± 56	124 ± 51	0.346
**Estimated GFR (ml/min) *(me8an±ds)***	47.5 ± 23.0	40.34 ± 20.9	**0.022**
**LVEF% *(mean±ds)***	47.1 ± 11.1	45.1 ± 10.9	0.207
**LAVI (ml/m2) *(mean±ds)***	32.8 ± 4.0	32.9 ± 4.1	0.933
**LVMI (g/m2) *(mean±ds)***	109.6 ± 17.9	107.7 ± 14.5	0.404
**CAD n (%) *(mean±ds)***	48(44.8)	40 (43.01)	0.887
**Chronic cerebrovascular disease n (%)**	25 (23.3)	12 (12.9)	0.069
**PAD n (%)**	16 (9)	9 (10.3)	0.290
**Chronic renal disease n (%)**	50 (46.7)	43 (46.2)	0.769
**Diabetes n (%)**	66 (61.6)	52 (55.9)	0.472
**Hypertension n (%)**	103 (96.2)	69 (55.9)	<0.0005
**Valvular heart disease n (%)**	33 (30.8)	27 (29.0)	0.877
**ischemic dilated cardiomyopathy (%)**	52 (48.6)	42 (45.2)	0.731
**non-ischemic dilated cardiomyopathy (%)**	17 (15.9)	14 (15.0)	0.973
**Atrial fibrillation n (%)**	45 (42.0)	28 (30.1)	0.105
**Anaemia n (%)**	20(18.6)	16 (17.2)	0.856
**Smoking n (%)**	38 (35.5)	25 (26.8)	0.223
**Rest dyspnoea n (%)**	24 (22.4)	21(22.5)	0.496
**work/effort dyspnoea**	88 (82.2)	77(82.6)	0.887
**Peripheral oedema n (%)**	99 (92.5)	88 (94.6)	0.802
**inappropriate drug reduction** **uncontrolled hypertension** **arrhythmias**	45 (42.0) 18 (16.8) 19 (17.7)	46 (49.4) 12 (12.9) 22 (23.6)	0.364 0.564 0.392
**NYHA Class** **II** **III** **IV**	24 (22.4) 64 (59.8) 19 (17.7)	20 (21.5) 56 (60.2) 17 (18.2)	0.989 0.931 0.929
**Mean daily dosage of intravenous furosemide (mg) *(mean±ds)***	156±22	160±26	
**Ace-inhibitors n (%)**	31 (28.9)	31 (33.3)	0.542
**ARBs n (%)**	34 (31.7)	26 (27.9)	0.643
**Beta blockers n (%)**	54 (50.4)	53 (56.9)	0.395
**MRA (%)**	17 (15.8)	24 (15.1)	0.113
**SGLT2 inhibitors (n/%)**	15 (14.0)	13 (13.9)	0.441
**Insulin (basal bolus) (n/%)**	36 (38.7)	34 (31.7)	0.766

Pts treated with i.v. furosemide plus HSS: patients randomized to i.v. high dosage furosemide plus small volume of hypertonic saline solutions (HSS); Pts treated with i.v. furosemide alone: patients randomized to i.v. high dosage furosemide alone; WBC: white blood cells; SBP, systolic blood pressure; DBP, diastolic blood pressure; BMI, body mass index; FPG, fasting plasma glucose; GFR, glomerular filtration rate; LVMI, left ventricular indexed mass; LAVI, left atrial volume index; LVEF, left ventricular ejection fraction; CAD, coronary artery disease; PAD, peripheral artery disease; LDL, low density lipoprotein; HDL, high density lipoprotein; NYHA, New York Heart Association; ARBs, angiotensin II receptor blocker; MRA, mineralcorticoid receptor antagonist.

Baseline, demographic and laboratory variables in patients randomized to receive i.v. furosemide plus HSS (Group A) and i.v. furosemide alone (Group B) group are reported in Table 1.

Subjects randomized to receive the furosemide + HSS showed at baseline a significantly higher frequency of hypertension (*96.2% vs 55.9%; p<0.0005*), higher systolic blood pressure (SBP) (*135.0±22.04 mm/Hg vs 120.5 ±13.8 mm/Hg; p<0.0005*), higher BMI (*28.5±5.6 kg/m^2^ vs 25.7±4.5 kg/m^2^; p<0.0005*), higher mean serum total cholesterol (*134.2±35.9 mg/dL vs 106.1±74.2 mg/dL; p=0.001*), higher mean serum triglycerides (*93.3±35.3 vs 64.9±26.4 mg/dL; p<0.0005*) in comparison to patients randomized to receive furosemide alone.

Following the treatment phase, the increase in urine output was greater in the furosemide + HSS group than in the furosemide-only group (1021.62±212.29 vs 2340.74±466.37 mL; p<0.0005 and 1004.57±146.70 vs 1907.35±269.36 mL; p<0.0005). In parallel, patients receiving i.v. furosemide + HSS experienced a more pronounced reduction in body weight compared with those treated with i.v. furosemide alone (73.8±5.2 vs 67.5±5.3; p<0.0005 and 72.9±4.1 vs 69.7±4.1; p<0.0005).

### Congestive HF symptoms in patients treated with i.v. furosemide + HSS vs i.v. furosemide alone

At baseline, 24 (22.4%) patients in the furosemide + HSS group and 21 (22.5%) in the furosemide-only group reported dyspnoea at rest; 88 (82.2%) and 77 (82.6%), respectively, complained of exertional dyspnoea; and 99 (92.5%) vs 88 (94.6%) reported peripheral oedema.

By T1, after the 6-day course of high-dose furosemide + HSS or furosemide alone, rest dyspnoea was present in 5 (4.6%; in-group p=0.013) patients in the furosemide + HSS group and in 4 (4.3%; in-group p=0.81) patients in the furosemide-only group (between-group p=0.24). Exertional dyspnoea persisted in 10 (9.3%; in-group p=0.0001) vs. 19 (20.4%; in-group p=0.0001) patients, respectively (between-group p=0.044). Peripheral oedema was still reported by 12 (11.21%; in-group p=0.0001) patients in the furosemide + HSS arm and by 21 (22.5%; in-group p=0.0001) in the furosemide-alone arm (between-group p=0.037).

### Serum inflammatory, stretch and myocardial stress markers after 6 days of i.v. furosemide + HSS vs i.v. furosemide alone

At baseline, there were no significant differences between groups in mean serum concentrations of IL-6, hsTnT, galectin-3 or NT-proBNP. In contrast, patients allocated to the furosemide + HSS regimen exhibited significantly higher baseline sST2 values compared with those receiving furosemide alone (41.6±8.8 pg/mL vs 37.5±6.9 pg/mL; between-group p<0.0005).

### “In-group” and “between group” analysis of inflammatory, stretching and myocardial stress marker serum levels changes after six-day treatment with i.v. furosemide + HSS vs treatment with i.v. furosemide alone.

At “*in-group” and “between group”* analyses, between T0 and T1, patients treated with intravenous moderate/high-dose furosemide plus HSS in comparison to control subjects treated with i.v. furosemide alone showed a more significant degree of reduction in the serum levels of IL-6 *(1.70± 0.82 vs. 2.31 ± 1.2 at in-group p <0.0005 and 1.88 ± 1.04 ± vs 2.04±08, p=0.170 at between group, respectively*), sST2 (*27.8± 7.7 vs. 41.6 ± 8.8 and 28.7±7.0 vs 37.5±6.9 at in-group p <0.0005 respectively, between group p =0.430)*, hsTnT (0.02 ± 0.01 vs 0.03±0.02 and 0.02±0.01 vs 0.03±0.02 *p<0.0005 at in-group respectively, p=0.692 at between group*), Galectin-3 (*11.6±1.9 vs 15.8±3.2 and 11.8±2.3 vs 16.6±3.8 at in-group p <0.0005 respectively, between group p =0.389*), NT-proBNP *(3279 ± 3963 vs 6786± 7110 and 4323± 4176 vs 5229±4571 at in-group p<0.0005 respectively, and p=0.071 at between group analysis).*

### “In-group” and “between group” analysis of epigenetic signatures changes (selected microRNA change fold) after six-day treatment with i.v. furosemide+ HSS vs treatment with i.v. furosemide alone.

**Figure 1:**
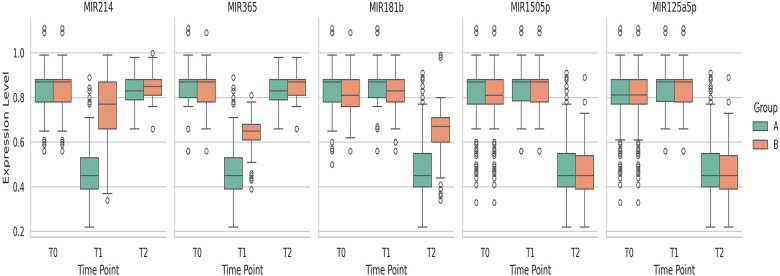
Distribution of MiRNA variables over time by group.

Regarding miRNA fold change, we observed at T1 at “between group” analysis only a significantly higher reduction of miR214 fold change in subjects treated with i.v. furosemide plus HSS in comparison to patients treated with i.v. furosemide alone *(0.47±0.12 vs 0.73±014; p <00005)* and of miR365 *(0.47±0.12 vs 0.63±0.07; p <00005)* (see Table 2) whereas we observed a more significant reduction of miR125a5p and of miR1505p* at T2 in subjects treated with furosemide alone in comparison to patients treated with i.v. furosemide plus HSS *(0.46±0.10 vs 0.51±0.16; p=0.033 and 0.46±0.11 vs 0.51±0.15; p=0.036)* and a more significant reduction of miRNA181b* fold in subjects treated with furosemide plus HSS in comparison to subjects treated with furosemide alone *(0.50± 0.15 vs 0.65±010; p<0.0005)* (see Figure 1).

**Table 2:** Myocardial stress variable values and micro-RNA fold changes, after furosemide + HSS or furosemide therapy at T(baseline), at T1 (after six days of treatment with i.v. furosemide+HSS or i.v. furosemide alone) and at T2 after saline load (after a saline load administered after the end of treatment with i.v. furosemide) in subjects with acute decompensated HFrEF.

**Variable**	**Groups**	**Admission-T 0**	**between-group p**		**T 1 -** **after 6 days of treatment**	**between-group p**		**In-group P (T0vsT1)**	**T2 -after acute saline load**	**between-group p**	**In-group p** **(T1vsT2)**
**IL6 (pg/ml)**	**furosemide alone group**	2.04 (0.8)	0.077	1.88 (1.04)			0.170	0.078	3.24 (1.18)	**<0.0005** **<0.0005§**	**<0.0005**
	**furosemide+HSS group**	2.31(1.2)		1.70(0.82)				**<0.0005**	1.96 (0.94)		**<0.0005**
**hsTnT (ng/ml)**	**furosemide alone group**	0.03 (0.02)	0.145	0.02(0.01)			0.692	**<0.0005**	0.03(0.03)	**<0.0005** **<0.0005§**	**<0.0005**
	**furosemide+HSS group**	0.03(0.02)		0.02(0.01)				**<0.0005**	0.01 (0.01)		**<0.0005**
**sST2 (ng/ml)**	**furosemide alone group**	37.5(6.9)	**<0.0005** **<0.0005§**	28.7(7.0)			0.430	**<0.0005**	43.6 (6.8)	**<0.0005** **<0.0005§**	**<0.0005**
	**furosemide+HSS group**	41.6(8.8)		27.8 (7.7)				**<0.0005**	36.9 (7.48)		**<0.0005**
**Gal 3 (ng/ml)**	**furosemide alone group**	16.60 (3.8)	0.130	11.8(2.3)			0.389	**<0.0005**	18.8 (2.92)	**<0.0005** **<0.0005§**	**<0.0005**
	**furosemide+HSS group**	15.8(3.2)		11.6(1.9)				**<0.0005**	13.8 (2.0)		**<0.0005**
**CRP (mg/dl)**	**furosemide alone group**	2.2 (0.7)	0.667	2.15(0.7)			0.366	1.0	2.3 (0.7)	0.079	0.719
	**furosemide+HSS group**	2.2 (0.8)		2.06(0.63)				0.173	2.1(0.6)		1.0
**NtPro BNP (pg/ml)**	**furosemide alone group**	5229 (4571)	0.072	4323(4176)			0.071	**<0.0005**	6083(5025)	**<0.0005** 0.343§	**<0.0005**
	**furosemide+HSS group**	6786 (7110)		3279(3963)				**<0.0005**	3195(3784)		**1.0**
**MIR214**	**furosemide alone group**	0.83(0.09)	0.785	0.73(0.14)			**<0.0005** **<0.0005§**	**<0.0005**	0.85(0.05)	0.107	**<**0.0005 <0.0005
	**furosemide+HSS group**	0.84(0.09)		0.47(0.12)				**<0.0005**	0.84(0.05)		
MIR365*	**furosemide alone group**	0.84(0.07)	0.266	0.63 (0.07)			**<0.0005** **<0.0005§**	**<0.0005**	0.85(0.05)	0.066	**<0.0005 <0.0005**
	**furosemide+HSS group**	0.85 (0.08)		0.47 (0.12)				**<0.0005**	0.84(0.05)		
MIR181b*	**furosemide alone group**	0.81(0.09)	0.203	0.82(0.08)			0.204	0.461	0.65 (0.10)	**<0.0005** **<0.0005§**	**<0.0005 <0.0005**
	**furosemide+HSS group**	0.83(0.10)		0.85(0.09)				**0.019**	0.50 (0.15)		
MIR1505p*	**furosemide alone group**	0.78(0.16)	0.702	0.85(0.09)			0.906	**<0.0005**	0.46(0.11)	**0.036** 1.0§	**<0.0005 <0.0005**
	**furosemide+HSS group**	0.79(0.15)		0.85 (0.09)				**<0.0005**	0.51 (0.15)		
MIR125a5p*	**furosemide alone group**	0.78(0.16)	0.774	0.85 (0.09)			0.989	**<0.0005**	0.46(0.10)	**0.033** 1.0§	**<0.0005 <0.0005**
	**furosemide+HSS group**	0.79(0.15)		0.85 (0.88)				**<0.0005**	0.51(0.16)		

HFrEF, heart failure with reduced ejection fraction; IL-6, interleukin-6; hsTnT, high sensitivity troponin-T; sST2, suppression of tumorigenicity 2; Gal 3, Galectin-3; CRP: C-reactive protein; NtPro BNP, N-terminal pro-brain natriuretic peptide; *MirNA fold increase in furosemide +HSS group and in Furosemide alone Group after six days of treatment. (T1) and after an acute saline load; §data adjusted for BMI, Hypertension, Total Cholesterol and triglycerides.

At “in-group” analysis, we observed a significant change at T0 vs T1 with regard to miR214 *(p<0.0005 in both groups),* miR365 (p<0.0005 in both groups), miR181b *(p=0.019 in furosemide plus HSS group),* miR1505p (p<0.0005 in both groups), miR125a5p. *(p<0.0005 in both groups)*.

### “In-group” and “between group” analysis of inflammatory, stretching and myocardial stress marker serum levels changes and epigenetic signatures changes (selected microRNA change fold) at T2 after acute saline load.

At “*in-group*” and *“between group”* analysis, at T2 after an acute saline load, controls treated with i.v. furosemide alone vs patients treated with intravenous moderate/high-dose furosemide plus HSS showed a significantly higher increase of serum levels of IL-6 *(1.96 ± 0.94 vs 1.70 ± 082 and 3.24±1.18 vs. 1.88 ± 1.04, p <0.0005 at between and in-group analysis, respectively),* sST2 (*43.6± 6.8 vs 28.7 ± 7.0 and 36.9±7.48 vs 27.8±7.7; between and in-group p <0.0005 respectively)*, hsTnT *(0.01 ± 0.01 vs 0.02±0.01 and 0.03±0.03 vs 0.02±0.01 between and in-group p <0.0005 respectively),* galectin-3 (*18.8±2.9 vs 11.8±2.3 and 13.8±2.0 vs 11.6±1.9 between and in-group p<0.0005 respectively)* NT-proBNP (*3195± 3784 vs 3279±3963 intra-group p=1 and 6083 ± 5025 vs 4323± 4176, p <0.0005 at intra-group analysis, between group, p<0.0005),* no significant change has been observed with regard of CRP serum levels (see Table 2).

At “*in-group*” and *“between group”* analysis, at T2 after an acute saline load, patients treated with intravenous moderate/high-dose furosemide plus HSS vs controls treated with i.v. furosemide alone showed a significantly higher fold change of miR214 *(0.84±0.05 vs 0.47±0.12 and 0.85±0.05 vs 0.73±0.14; in-group p<0.0005 respectively and between group p=0.107),* miR365 *(0.84±0.05 vs 0.47±0.12 and 0.85±0.05 vs 0.63±0.07; in-group p <0.0005 respectively and between group p=0.66)* and a significantly lower fold change of miR181b (*0.50±0.15 vs 0.85±0.09 and 0.65±0.10 vs and 0.82±0.08; between group p <0.0005 and in-group p<0.0005).* Controls treated with furosemide alone in comparison to patients treated with intravenous moderate/high-dose furosemide plus HSS showed a significantly lower fold change of miR125a5p *(0.46±0.10 vs 0.85±0.09 and 0.51±0.16 vs and 0.85±0.88; between group p=0.033 and in-group p<0.0005)*.

### Analysis of absolute delta (Δ) values of inflammatory, stretching and myocardial stress marker serum levels at T1 after six-day treatment with i.v. furosemide+ HSS vs treatment with i.v. furosemide alone and at T2 after acute saline load.

When comparing the absolute within-group delta (Δ) values at T0–T1—defined as the difference in serum biomarker levels between baseline (T0) and after the treatment phase with furosemide + HSS or furosemide alone (T1)—we found that patients receiving high-dose furosemide + HSS exhibited significantly greater Δ reductions in IL-6 (0.60±0.78 vs 0.16±0.69 pg/mL; p<0.0005), sST2 (13.7±8.34 vs 8.8±4.2; p<0.0005) and NT-proBNP (3506±4594 vs 905±1950; p<0.0005) compared with those treated with furosemide alone (Table 3). In contrast, no significant between-group differences were observed for within-group Δ(T0–T1) values of hsTnT, galectin-3 or CRP (Table 3).

**Table 3:** Absolute delta values (Δ) in case and controls at T1 (T0-T1) and T2 time (T1-T2) in subjects treated with with i.v. furosemide+ HSS or i.v. furosemide alone.

**Variable**	**Groups**	**DELTA 0-1**	**Between- group p**	**DELTA 0-2**	**Between-group p**	**DELTA 1-2**	**Between-group p**
**Δ IL-6**	**furosemide alone group**	0.16 (0.69)	**<0.0005** 0.06§	-1.19 (0.10)	**<0.0005** **<0.0005§**	-1.35 (0.92)	**<0.0005** **<0.0005§**
	**furosemide+HSS group**	0.60 (0.78)		0.35(0.69)		-0.25 (0.41)	
**Δ hsTnT**	**furosemide alone group**	0.01(0.02)	0.081	-0.005(0.02)	**<0.0005** **0.002§**	-0.01(0.02)	**<0.0005** **<0.0005§**
	**furosemide+HSS group**	0.007 (0.12)		0.002 (0.01)		0.00 (0.0)	
**Δ sST2**	**furosemide alone group**	8.8 (4.22)	**<0.0005** **<0.0005§**	-6.16 (6.03)	**<0.0005** **<0.0005§**	-14.9 (5.97)	**<0.0005** **<0.0005§**
	**furosemide+HSS group**	13.7 (8.34)		4.67 (7.72)		-9.03 (5.9)	
**Δ Gal 3**	**furosemide alone group**	4.7 (4.10)	0.322	-2.17 (3.92)	**<0.0005** **<0.0005§**	-6.94 (3.4)	**<0.0005** **<0.0005§**
	**furosemide+HSS group**	4.3 (2.9)		2.08 (3.2)		-2.19 (1.43)	
**Δ CRP** (mg/dl)	**furosemide alone group**	0.05(0.85)	0.651	-0.06(0.96)	0.305	-0.15(0.91)	0.424
	**furosemide+HSS group**	0.014(0.49)		0.1(0.74)		-0.04(0.60)	
**Δ NtPro BNP** (pg/ml)	**furosemide alone group**	905 (1950)	**<0.0005** **<0.0005§**	-854 (1239)	**<0.0005** **<0.0005§**	-1760(2114)	**<0.0005** **<0.0005§**
	**furosemide+HSS group**	3506(4594)		3591(4780)		84.2(1487)	

IL-6, interleukin-6; hsTnT, high sensitivity troponin-T; sST2, suppression of tumorigenicity 2; Gal 3, Galectin-3; CRP, C-reactive protein; NtPro BNP, N-terminal pro-brain natriuretic peptide; §data adjusted for BMI, Hypertension, Total Cholesterol and triglycerides.

We also analyzed between-group differences in absolute Δ values at T1–T2, representing the change in serum biomarker levels between the end of the treatment period (T1) and after the acute saline load (T2). Patients treated with furosemide + HSS showed significantly higher Δ(T1–T2) values for IL-6 (−0.25±0.41 vs −1.35±0.92 pg/mL; p<0.0005), hsTnT (0.00 vs −0.01±0.02; p<0.0005), sST2 (−9.03±5.9 vs −14.9±5.97; p<0.0005), NT-proBNP (84.2±1487 vs −1760±2114; p<0.0005) and galectin-3 (−2.19±1.43 vs −6.94±3.4; p<0.0005) compared with the furosemide-only group (Table 3). No significant between-group difference was detected for Δ(T1–T2) CRP values (Table 3).

Regarding circulating miRNAs, the analysis of within-group absolute Δ(T0–T1) values—reflecting the change in fold expression between baseline (T0) and post-treatment (T1)—showed that patients receiving high-dose furosemide + HSS had significantly greater Δ fold changes in miR-214 (0.36±0.14 vs 0.09±0.17; p<0.0005), miR-365 (0.38±0.13 vs 0.20±0.10; p<0.0005) and miR-181b (−0.02±0.08 vs −0.01±0.09; p<0.0005), compared with those treated with furosemide alone (Table 4).

**Table 4:** Absolute delta of MirNA fold increase in furosemide +HSS group and in furosemide alone group after six days of treatment. (delta =-1) and after an acute saline load (delta 1-2).

**Variable**	**Groups**	**DELTA 0-1**	**Between- group p**	**DELTA 0-2**	**Between-group p**	**DELTA 1-2**	**Between-group p**
**MIR214**	**furosemide alone group**	0.09 (0.17)	**<0.0005** **<0.0005§**	-0.15 (0.11)	0.349	-0.11 (0.15)	**<0.0005** **<0.0005§**
	**furosemide+HSS group**	0.36 (0.14)		0.001(0.12)		-0.36 (0.12)	
MIR365*	**furosemide alone group**	0.20 (0.10)	**<0.0005** **<0.0005§**	-0.01(0.09)	0.067	-0.21 (0.09)	**<0.0005** **<0.0005§**
	**furosemide+HSS group**	0.38 (0.13)		0.016 (0.10)		-0.36(0.12)	
MIR181b*	**furosemide alone group**	-0,01 (0.09)	**<0.0005** **<00005§**	0,16 (0.14)	0.485	0.16 (0.13)	**<0.0005** **<0.0005§**
	**furosemide+HSS group**	-0,02 (0.08)		0.33 (0.17)		0.35 (0.16)	
MIR1505p*	**furosemide alone group**	-0.06 (0.13)	0.766	0.31 (0.17)	0.215	0.38 (0.12)	0.068
	**furosemide+HSS group**	-0.06 (0.13)		0.28 (0.19)		0.34 (0.16)	
MIR125a5p*	**furosemide alone group**	-0.06 (0.13)	0.737	0.32 (0.17)	0.148	0.38 (0.12)	0.051
	**furosemide+HSS group**	-0.05 (0.13)		0.28 (0.19)		0.34 (0.16)	

MIR214 : microRNA 214; MIR365* : microRNA 365; MIR181b*: microRNA 181b; MIR1505p*: microRNA1505p; MIR125a5p*: microRNA 125a5p*; § data adjusted for BMI, Hypertension, Total Cholesterol and triglycerides.

**Figure 2:**
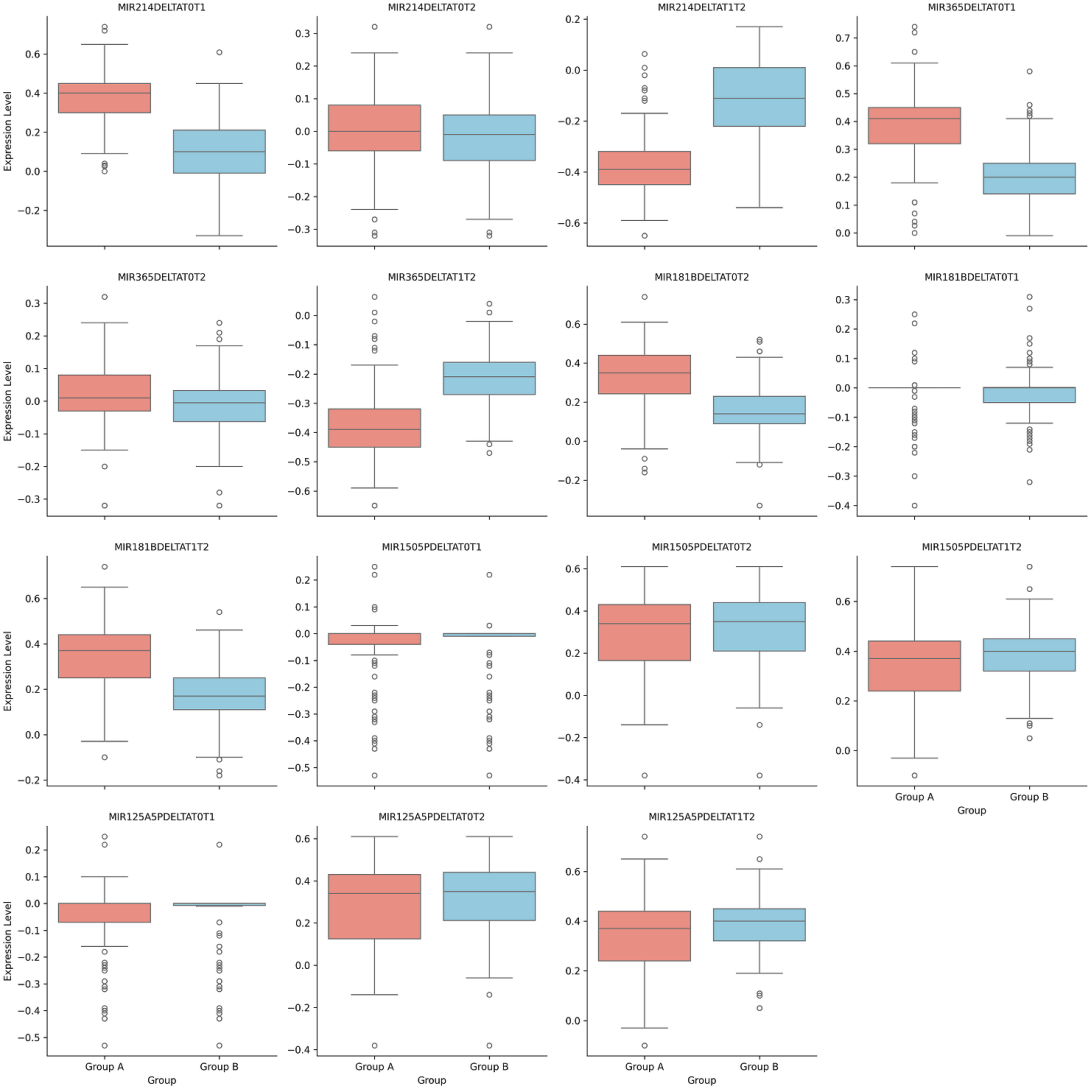
Delta changes at different time for analyzed MiRNAs.

Moreover, significantly larger absolute Δ(T1–T2) values were observed within groups when comparing the end of treatment (T1) to the post–acute saline load phase (T2) for miR-214 (−0.36±0.12 vs −0.11±0.15; p<0.0005), miR-365 (−0.36±0.12 vs 0.21±0.09; p<0.0005) and miR-181b (0.35±0.16 vs 0.16±0.13; p<0.0005) in the furosemide + HSS group relative to the furosemide-only group (Table 4 and Figure 2).

## DISCUSSION

Our data indicate that, in patients with ADHF and HFrEF, treatment with moderate-to-high doses of i.v. furosemide in combination with HSS, compared with furosemide alone, is associated with a significantly greater reduction in circulating levels of IL-6, sST2, hsTnT, galectin-3 and NT-proBNP.

In a previous study of 94 patients with refractory chronic HF who received i.v. furosemide (500–1,000 mg) plus HSS twice daily, investigators reported a marked increase in daily diuresis and natriuresis, a more rapid decline in BNP concentrations, shorter hospital stays and a lower 30-day readmission rate [38].

In another recent study from our group [39], we observed a comparable magnitude of reduction in three key HF biomarkers, suggesting that the decrease in circulating HF markers is not solely attributable to more pronounced decongestion or faster attainment of clinical compensation. This observation raises the possibility that the therapeutic benefits of this regimen may extend beyond simple relief of congestion-related signs and symptoms. In line with this, the higher delta values documented after treatment with i.v. furosemide plus HSS in the present study point to a potentially greater biological efficacy, possibly mediated through more favourable modulation of myocardial stretch and fibrotic pathways.

Overall, our findings are consistent with prior reports showing superior clinical improvement with i.v. furosemide + HSS compared with furosemide alone, although relatively few studies have specifically evaluated the impact of this strategy on inflammatory and fibrosis-related HF biomarkers [21, 25, 26, 38].

It is well established that a variety of biologically active mediators, including cytokines, are upregulated in the context of HF [36, 37, 40]. In particular, circulating TNF-α and IL-6 levels are elevated and correlate with HF severity [9]. Previous work has demonstrated that optimization of conventional HF therapy with diuretics, ACE inhibitors, beta-blockers and digoxin can significantly reduce serum TNF and IL-6 concentrations [41].

Among the numerous emerging biomarkers, sST2 has been identified as one of the most promising in recent investigations. ST2, a member of the interleukin-1 receptor family also known as interleukin-1 receptor-like 1 (IL1RL1), has been reported to be expressed by cardiac cells in response to myocardial stress [42–45].

Over the past decade, several studies have measured soluble ST2 in different HF settings, generating robust and reproducible evidence for its prognostic value, both additive to and independent of established markers [46–48]. Alternative splicing of the ST2 gene gives rise to multiple isoforms, including a transmembrane receptor (ST2L) and a soluble circulating form (sST2, often simply referred to as ST2), which has gained particular relevance as a biomarker. Both ST2L and sST2 are produced by cardiomyocytes and cardiac fibroblasts in response to mechanical stress, and both bind interleukin-33 (IL-33). IL-33 itself is induced by cellular stretch and appears to exert anti-fibrotic and anti-hypertrophic effects in mechanically stressed tissues through activation of MyD88 (myeloid differentiation primary response 88), IRAK (interleukin-1 receptor-associated kinase), ERK (extracellular signal-regulated kinase) and, ultimately, NF-κB (nuclear factor-κB).

Several studies have shown that the combination of furosemide with HSS is more effective than furosemide alone in improving congestion. Achieving adequate systemic decongestion while preserving or improving renal function is a central goal in HF management [34–40, 49]. When quantified by the degree of haemoconcentration, more complete decongestion has been associated with lower all-cause and cardiovascular mortality and reduced rehospitalization, even in the presence of transient worsening renal function [17, 21, 25, 50, 51]. Beyond this, the addition of HSS to i.v. furosemide may have favourable pleiotropic effects on cardiac remodelling and inflammatory pathways that characterize the congestive HF syndrome [52–56].

Some reports have suggested that furosemide combined with HSS can protect renal function, thereby enhancing diuretic response and improving clinical outcomes [51, 57]. However, the overall efficacy of HSS plus furosemide in acute HF remains debated. Other studies have found comparable effects between combined HSS–furosemide therapy and furosemide alone, with no significant differences in serum creatinine, body weight or urine output [58, 59].

Extracellular volume expansion is a fundamental pathophysiological feature of HF. Excess extracellular fluid leads to elevated intracardiac filling pressures, which in turn give rise to the cluster of signs and symptoms collectively referred to as congestion. Loop diuretics remain a mainstay of HF therapy, yet, unlike other HF treatments, high-quality randomized trial evidence to guide their optimal use is limited. Effective and safe diuretic management in both inpatient and outpatient settings requires a sophisticated understanding of renal physiology and diuretic pharmacokinetics. Diuretic resistance—defined as an inadequate natriuretic response despite appropriate diuretic dosing—represents a major therapeutic challenge and is generally associated with an adverse prognosis [60, 61].

A recent meta-analysis reported that HSS combined with furosemide increases 24-hour urine output and reduces body weight and serum creatinine in patients with acute HF. Furthermore, patients receiving HSS plus furosemide had lower rates of rehospitalization and mortality compared with those treated with furosemide alone [60].

In our cohort, we also observed that, after attaining clinical compensation and undergoing an acute volume challenge with a rapid i.v. saline load, both patients treated with high-dose furosemide plus HSS and those receiving furosemide alone experienced increases in serum IL-6, sST2 and galectin-3, but not in NT-proBNP. Notably, the magnitude of the increase in IL-6, sST2 and galectin-3 was significantly smaller in the group treated with i.v. furosemide + HSS than in the furosemide-only group, further supporting a more favourable modulation of inflammatory and remodelling pathways with the combined regimen. Some previous studies sustain the use of an isotonic saline load to challenge clinical compensation in patients with ventricular dysfunction [59–61].

In our previous work [32], we also found that, following an acute saline load, patients treated with high-dose i.v. furosemide plus HSS exhibited a significantly smaller percentage change in natriuretic peptides and immuno-inflammatory markers compared with control groups.

Several studies have examined ANP and BNP responses to acute saline infusion. Langer et al. [62] reported that an i.v. saline bolus (18 mL/kg sodium chloride at 250 mL/min) in healthy men led to a rise in plasma ANP concentrations without a measurable increase in plasma BNP for at least 60 minutes after the load. In contrast, Heringlake et al. [63], using i.v. volume expansion with 15 mL/kg of 0.9% NaCl over 60 minutes, showed that saline infusion induced a brief elevation in plasma NT-pro-ANP followed by a delayed increase in NT-pro-BNP. These and other investigations were all performed in healthy subjects [62, 63]. By comparison, Volpe et al. [64] demonstrated that plasma ANF levels do not rise in response to volume expansion in patients with dilated cardiomyopathy and mild ventricular dysfunction.

Taken together, these studies indicate that an acute saline load in clinically stable individuals is safe and provides a useful tool to probe abnormalities in neurohormonal responses to atrial stretch [62–64]. Experimental data further show that hemodynamic pressure overload provokes a pronounced, though transient, upregulation of proinflammatory cytokines and cytokine receptor genes in the adult heart, supporting the concept that cytokine expression is at least partly load-dependent. Consistent with this notion, our findings suggest that volume depletion induced by high-dose furosemide leads to a marked reduction in circulating cytokine levels that is only partially reversed by subsequent acute volume loading [32].

Collectively, these studies indicate that an acute saline load in clinically stable individuals is safe and represents a useful tool to probe abnormalities in neurohormonal responses to atrial distension [62–64]. Hemodynamic pressure overload has been shown to trigger a marked, albeit transient, upregulation of proinflammatory cytokine and cytokine receptor gene expression in the adult mammalian heart, supporting the concept that cytokine expression is, at least in part, load-dependent. This notion is in line with our findings, which show that volume depletion induced by high-dose furosemide results in a significant reduction in circulating cytokine levels that is only partially reversed by subsequent acute volume loading.

The attenuated percentage change in selected biomarker levels after saline loading in patients treated with high-dose furosemide plus HSS is not straightforward to explain. One plausible hypothesis is that high-dose furosemide combined with HSS, by reducing volume overload and rapidly increasing extracellular NaCl concentration, may relieve myocardial stretch and thereby modulate circulating natriuretic peptides and immuno-inflammatory markers, although the underlying mechanisms remain speculative. To date, no study has specifically examined the effect of HSS on intracellular sodium ([Na^+^]) and its relationship with natriuretic peptide biology. Nonetheless, it is conceivable—pending further experimental and clinical work—that a beneficial effect of HSS on myocardial electrolyte homeostasis may contribute to metabolic protection of the myocardium.

Although the precise mechanism of action of HSS is still not fully defined, several hypotheses have been proposed. Furosemide exerts its diuretic effect after active secretion into the tubular lumen from proximal tubular cells. Many patients with ADHF develop intravascular hypovolaemia and reduced renal blood flow, which impairs this secretion process [48]. Administration of HSS has been shown to increase intraluminal furosemide concentrations and to augment 24-hour diuresis, urinary sodium excretion and urinary osmolality [62, 63]. In addition, HSS has been associated with reductions in plasma renin activity and ANP levels [64].

In the present study, at the compensated stage achieved by both treatment arms, we observed that, after acute saline loading, patients receiving i.v. furosemide + HSS, compared with those treated with furosemide alone, displayed a significantly smaller increase in serum IL-6, hsTnT, sST2, galectin-3 and NT-proBNP. Only a limited number of studies to date have evaluated the impact of i.v. diuretic therapy on neurohormonal and immuno-inflammatory biomarkers. In our setting, the reduction in IL-6 and other markers can reasonably be ascribed to the addition of HSS, which appears to potentiate the effect of furosemide. The transient rise in biomarkers with hemodynamic overload supports the notion that significant cardiomyocyte stretch is occurring. High-dose furosemide plus HSS, by mitigating volume overload and rapidly increasing extracellular NaCl concentrations, may reduce cardiomyocyte stretch and thereby influence NP and immune-inflammatory serum markers [26].

Accordingly, our observation of a less pronounced within-group increase in IL-6, sST2 and NT-proBNP after an acute saline load in patients treated with high-dose furosemide plus HSS, compared with those receiving furosemide alone, is likely related to modulation of the myocardial stretch response to volume overload. We further noted that, at T2 (after saline loading), patients treated for 6 days with i.v. furosemide + HSS showed an increase in IL-6, sST2 and galectin-3 levels relative to T1 (end of i.v. therapy), but these concentrations remained below baseline (T0) values. No significant changes in CRP or hsTnT were detected.

It should also be acknowledged that the higher mean systolic blood pressure observed in the furosemide/HSS group may represent a potential confounder for our results.

The heart is a remarkably plastic organ, capable of undergoing extensive structural and functional remodelling in response to diverse environmental and pathophysiological stimuli [60]. Parallel to these remodelling processes, substantial transcriptional reprogramming takes place in the failing myocardium, and the pivotal contribution of epigenetic regulatory mechanisms in shaping these events has been well recognized [61]. Key components of the epigenetic machinery are biochemically coupled to cellular metabolism through their dependence on specific substrates and cofactors for enzymatic activity [62]. For instance, one study demonstrated that chromatin acetylation machinery can sense alterations in cellular metabolism via acetyl-CoA availability and translate them into transcriptional changes that drive robust phenotypic shifts in pluripotent stem cells [63]. Other biological systems, including stem cells, exhibit similar epigenetic control, whereby a relatively simple metabolic switch can induce widespread genetic reprogramming [64].

Because epigenetic mechanisms are exquisitely sensitive to cellular energetics and mitochondrial function, the epigenetic landscape of the heart under conditions of metabolic and ageing stress undergoes profound changes. From a therapeutic standpoint, these targetable regulators of gene expression hold considerable promise. Indeed, pharmacological modulation of epigenetic modifiers has shown beneficial effects on pathological cardiac remodelling, with reported actions on inflammation, fibrosis, ischaemic injury and hypertrophy [60].

Given that miRNAs can regulate a broad spectrum of target mRNAs, and that individual genes may be modulated by multiple miRNAs, the intricate communication network between these molecules enables miRNAs to influence numerous biological processes, including hypertrophy, fibrosis and angiogenesis, among others. Consequently, miRNAs have substantial potential as therapeutic tools to prevent, attenuate or even reverse cardiac dysfunction, including adverse ventricular remodelling.

In this context, we also documented a more pronounced modulation of HF-related epigenetic signatures, as reflected by changes in selected microRNA fold expression among patients randomized to high-dose furosemide + HSS compared with those receiving i.v. furosemide alone. Specifically, after treatment with high-dose furosemide + HSS, we observed a greater reduction in the fold change of miR-214 and miR-181b* in the combination group than in the furosemide-only group, whereas at T2 a more marked decrease in miR-125a-5p and miR-150-5p* was evident in patients treated with furosemide alone than in those receiving i.v. furosemide + HSS.

MiR-214 has been implicated as a critical regulator of cardiac hypertrophy and maladaptive remodelling pathways involved in HF pathophysiology [65]. Decreased myocardial miR-214 expression has been reported in patients with established cardiac hypertrophy [66, 67]. Moreover, a recent study showed that individuals with systolic HF treated with carvedilol, compared with untreated counterparts, exhibited a significant increase in miR-214 expression [68].

The miR-181 family comprises four members—miR-181a, miR-181b, miR-181c and miR-181d. Although miR-181b has frequently been characterized as an oncogenic miRNA, it has also been associated with vascular inflammatory responses, supporting its role as a modulator of inflammation in HF [69].

MicroRNA-125a-5p (miR-125a-5p) is highly enriched in mesenchymal stromal cell–derived exosomes (MSC-Exos). In a murine I/R model, intramyocardial delivery of miR-125a-5p agomirs, MSCs or MSC-Exos conferred robust cardioprotection, improving cardiac performance and attenuating adverse remodelling. Treatment with miR-125a-5p agomir promoted M2 macrophage polarization, enhanced angiogenesis and reduced fibroblast proliferation and activation, ultimately limiting cardiomyocyte apoptosis and inflammatory responses [68]. Circulating miR-125a-5p has also been proposed as a biomarker of endothelial dysfunction and has been linked to the development of cardiomyopathy [65]. Its expression increases at the onset of reperfusion following ischaemia, where it appears to exert cardioprotective effects by facilitating nitric oxide (NO) signalling [67]. Whether miR-125a-5p mediates MIR4435-2HG–dependent regulatory mechanisms in cardiovascular disease remains to be clarified.

Overall, circulating microRNAs represent highly attractive epigenetic biomarkers because of their biological stability and ease of detection in liquid biopsies. Given the complexity of microRNA-mediated regulation of gene expression and post-transcriptional modification, disease-associated circulating miRNA signatures may mirror underlying pathophysiological states, offering valuable information for distinguishing specific subtypes and stages of complex cardiovascular conditions.

Nevertheless, microRNA serum levels could be considered not only HF diagnostic markers but may also have a possible role as therapeutic indicators.

A prospective, non-randomized self-control trial was performed in 81 patients with HF eligible for CRT to analyze the HF miRNA profile, reporting that HF patients were characterized by lower expression compared to control patients [23]. HF patients considered responders to CRT showed an increase in expression of 19 miRNAs compared with baseline expression, whereas in the non-responders, we observed an increase of six miRNAs compared with baseline expression.

Our findings indicate how the treatment protocol adopted in our study is associated with a higher degree of reduction of change fold of some of the chosen miRNA that have been reported as involved in some of the crucial pathogenetic steps of heart dysfunction such as ventricular hypertrophy and inflammation.

Thus, we have reported that our treatment protocol with high dose i.v. furosemide plus HSS is also characterized by some positive effects also in terms of epigenetic signatures indicating that this type of treatment seems to have on the remodeling mechanisms of the pathogenetic cascade linking to acute systolic left ventricular dysfunction.

miRNAs are involved in the regulation of central elements of the cardiac remodelling cascade, including cardiomyocyte biology and survival, extracellular matrix turnover and neurohormonal signalling. Because miRNAs are coordinately regulated in response to diverse stress stimuli and, in turn, modulate networks of genes that shape the “heart failure phenotype”, it is plausible that individual miRNAs, or specific miRNA clusters, govern the shift from adaptive to maladaptive cardiac remodelling [69–72].

In our cohort, we also detected significantly larger within-group delta (Δ) values at T1–T2—defined as the difference between post-treatment (T1) and post–acute saline load (T2) levels—for miR-21, miR-365 and miR-181b in patients treated with furosemide + HSS or furosemide alone.

MiR-21 is a 22-nucleotide microRNA that binds target mRNAs via complementary base pairing and regulates gene expression by promoting mRNA degradation or inhibiting translation. It has been linked to several forms of cardiomyopathy, including HF, dilated cardiomyopathy, myocardial infarction and diabetic cardiomyopathy.

In these settings, miR-21 expression is markedly altered both in the myocardium and in the circulation, where it appears to confer a degree of cardioprotection following injury, while at the same time being tightly linked to maladaptive processes such as cardiac hypertrophy and fibrosis [70].

A close relationship exists between cardiomyocyte autophagy and the development of cardiac hypertrophy, and persistent stress can drive hypertrophy to progress towards overt HF. MicroRNA-365 (miR-365) has been identified as a positive regulator of cardiac hypertrophy. In a recent study [69], miR-365 expression was found to be upregulated in hypertrophic cardiomyocytes *in vivo* and *in vitro*, in parallel with autophagy dysregulation, and miR-365 was shown to negatively modulate autophagic activity in these cells. Collectively, these data support a functional role for miR-365 in the development of cardiac hypertrophy and its transition to systolic dysfunction.

Acute myocardial stretch induced by hemodynamic overload is one of the most common stressors faced by the heart, and the capacity to mount an intrinsic adaptive response is crucial to preventing circulatory congestion. Mechanical stretch stimulates cardiomyocytes to secrete the cardiac natriuretic peptides ANP and BNP, which exert their effects by activating particulate guanylate cyclase A at the cell surface and increasing subsarcolemmal cGMP concentrations [70–75]. The myocardial response to acute stretch thus represents a fundamental adaptive mechanism.

Within this framework, our observation of a blunted rise in inflammatory markers and indices of myocardial stretch after an acute saline load in patients treated with HSS plus moderate/high-dose furosemide suggests that this combination may exert a modulatory effect on these pathways in HFrEF. Such an effect could have important clinical implications, indicating therapeutic benefits that extend beyond mere improvement in congestion-related symptoms and signs. Indeed, the association of i.v. furosemide with HSS may contribute to a “de-remodelling” effect, particularly in terms of attenuating atrial stretch and fibrosis.

This hypothesis could be further sustained and enhanced by our finding related to miRNA change fold after an acute saline load with higher degree of change fold of three miRNA after saline load in controls in comparison to patients treated with high dose furosemide plus HSS further indicating an epigenetic signature of this de-remodeling effect linked to our protocol treatment.

### Limitations

This study has several limitations. First, the sample size is relatively modest and would benefit from further expansion. Second, the administered furosemide doses were not uniform across patients, and the cohort included heterogeneous HF etiologies (ischemic, dilated, valvular, etc.). Ongoing recruitment and future analyses stratified by type of decompensation and underlying pathogenesis are therefore warranted. In addition, at baseline, patients in the furosemide + HSS group, compared with those in the furosemide-only group, exhibited a higher prevalence of hypertension, higher systolic blood pressure, greater BMI, as well as higher mean total cholesterol and triglyceride levels, which may represent potential confounders.

Another possible limitation is due to the fact that since the design of the study the hypertonic saline solution effects can’t be completely isolated. In future studies addressing this issue we wonder to add furosemide plus normal saline (volume control) and/or half-normal saline (intermediate osmolality control) in order to better evaluate the effects of HSS.

A further limitation is that the variability of the dosage is in the indicated cumulative dosing but it was not possible to address this variability analytically and this may represent a potential limitation.

Another potential limitation is the higher baseline sST2 concentrations (T0) observed in patients randomized to i.v. furosemide + HSS compared with those assigned to i.v. furosemide alone. In addition, the considerable variability in i.v. furosemide dosing within both treatment arms makes it difficult to disentangle and precisely quantify the isolated impact of furosemide dose on congestive HF biomarkers after the treatment periods.

Furthermore, the individualized furosemide dosing (120-250 mg bid) remains unadjusted; without stratification or regression thus conclusions about biomarker modulation are vulnerable to dosing bias and this may represent a possible limitation of the study.

## CONCLUSIONS

Our results demonstrate that, in patients with ADHF secondary to HFrEF, treatment with moderate-to-high doses of i.v. furosemide in combination with HSS is associated with a significantly greater reduction in circulating IL-6, sST2, hsTnT, galectin-3 and NT-proBNP levels.

Furthermore, we observed that, following an acute saline load, patients receiving high-dose furosemide plus HSS exhibited a smaller rise in several HF-related serum biomarkers, as reflected by lower delta values for the differences in their concentrations before and after volume challenge.

### Availability of data and material

The database is available on figshare (https://doi.org/10.6084/m9.figshare.28601864.v1).
